# The trauma patient in hemorrhagic shock: how is the C-priority addressed between emergency and ICU admission?

**DOI:** 10.1186/1757-7241-20-78

**Published:** 2012-12-03

**Authors:** Sigune Peiniger, Thomas Paffrath, Manuel Mutschler, Thomas Brockamp, Matthew Borgmann, Philip C Spinella, Bertil Bouillon, Marc Maegele

**Affiliations:** 1 Department of Trauma and Orthopedic Surgery, University of Witten/Herdecke, Cologne-Merheim Medical Centre (CMMC), Ostmerheimerstr.200, Cologne, D-51109, Germany; 2 Institute for Research in Operative Medicine (IFOM), University of Witten/Herdecke, Cologne-Merheim Medical Center (CMMC), Ostmerheimerstr.200, Cologne, D-51109, Germany; 3 San Antonio Military Medical Center, 3851 Roger Brooke Drive, San Antonio, TX, 78234, USA; 4 Division of Pediatric Critical Care, Washington University in St Louis, St Louis Children’s Hospital, One Children’s Place Suite 5820, Saint Louis, MO, 63110, USA

**Keywords:** Physiology, Coagulopathy, Fresh frozen plasma, Packed red blood cells, Platelets count, Shock, Adult, Severely injured, Trauma

## Abstract

**Background:**

Trauma is the leading cause of death in young people with an injury related mortality rate of 47.6/100,000 in European high income countries. Early deaths often result from rapidly evolving and deteriorating secondary complications e.g. shock, hypoxia or uncontrolled hemorrhage. The present study assessed how well ABC priorities (A: Airway, B: Breathing/Ventilation and C: Circulation with hemorrhage control) with focus on the C-priority including coagulation management are addressed during early trauma care and to what extent these priorities have been controlled for prior to ICU admission among patients arriving to the ER in states of moderate or severe hemorrhagic shock.

**Methods:**

A retrospective analysis of data documented in the TraumaRegister of the ‘Deutsche Gesellschaft für Unfallchirurgie’ (TR-DGU®^)^ was conducted. Relevant clinical and laboratory parameters reflecting status and basic physiology of severely injured patients (ISS ≥ 25) in either moderate or severe shock according to base excess levels (BE -2 to -6 or BE < -6) as surrogate for shock and hemorrhage combined with coagulopathy (Quick’s value <70%) were analyzed upon ER arrival and ICU admission.

**Results:**

A total of 517 datasets was eligible for analysis. Upon ICU admission shock was reversed to BE > -2 in 36.4% and in 26.4% according to the subgroups. Two of three patients with initially moderate shock and three out of four patients with severe shock upon ER arrival were still in shock upon ICU admission. All patients suffered from coagulation dysfunction upon ER arrival (Quick’s value ≤ 70%). Upon ICU admission 3 out of 4 patients in both groups still had a disturbed coagulation function. The number of patients with significant thrombocytopenia had increased 5-6 fold between ER and ICU admission.

**Conclusion:**

The C-priority including coagulation management was not adequately addressed during primary survey and initial resuscitation between ER and ICU admission, in this cohort of severely injured patients.

## Background

Worldwide, an estimated 5 million people died due to traumatic injuries in 2000 [1]. Nearly 50% of the world injury related death occurs in young people aged between 15-44 years with their productive years ahead. In European high income countries the injury related mortality rate was 47.6/100,000 population [1]. Death from trauma usually occurs early and over one-third of all in-hospital trauma deaths occur within the first six hours according to data from two large European trauma datasets [2]. While the immediate deaths after trauma are usually due to apnoea, severe brain or spinal cord injury or large vessel rupture, early deaths often result from rapidly evolving and deteriorating secondary complications such as shock, hypoxia, respiratory failure or uncontrolled hemorrhage. It has frequently been shown that early detection and aggressive management of complications secondary to trauma may improve survival and outcome for example via early damage control resuscitation [3-5]. To date, the Advanced Trauma Life Support (ATLS) has been implemented widely as a standard of care for initial assessment and treatment in trauma centres on the premise to “treat first what kills first”. This program identifies A: airway maintenance; B: breathing/ventilation; C: circulation with hemorrhage control; D: disability and E: exposure/environmental control as key issues to address during primary survey and treatment and suggests a simple mnemonic, e.g. ABCDE, as a memory trigger in which order the major problems upon emergency room (ER) arrival should be addressed. The present study assessed in how far ABC priorities with focus on C-priority including coagulation management are addressed during early in-hospital care and to what extent basic physiology has been restored prior to ICU admission among patients arriving to the ER in states of moderate or severe hemorrhagic shock.

## Methods

A retrospective analysis of data from severely injured patients documented in the TraumaRegister of the ‘Deutsche Gesellschaft für Unfallchirurgie’ (TR-DGU®) was conducted.

### TraumaRegister DGU®

The TraumaRegister DGU® (TR-DGU®) was founded in 1993 by the German Society for Trauma Surgery (Deutsche Gesellschaft für Unfallchirurgie, DGU®). It is a prospective, multicentre, standardized and anonymous documentation of multiple injured trauma patients at four consecutive post-trauma phases from injury to hospital discharge: (i.) the pre-hospital phase; (ii.) emergency room and initial surgery; (iii.) intensive care unit (ICU) and (iv.) outcome status at discharge and description of injuries and procedures. Between 01.01.2002 and 31.12.2008, 31,124 patients have been entered into the registry with 116 hospitals contributing data into the database. Hospitals affiliated with the TR-DGU® are mostly level-I and level-II trauma centers. Approximately 25% of all trauma patients in Germany are captured by the TR-DGU®. All injuries entered into the registry are coded using the Abbreviated Injury Scale (AIS). The trauma registry is approved by the review board of the German Society of Trauma Surgery (DGU) and is in compliance with the institutional requirements. As the TR-DGU® is an anonymous registry the Institution Review Board has waived no need for informed consent. In general, pre-hospital care in Germany is provided by a physician staffed emergency medical service (EMS).

### Study cohort

Inclusion criteria for the present analysis were age ≥18 years, primary admission, blunt trauma mechanism, survival prior to ICU admission, severe injury (ISS ≥ 25) in the absence of severe head trauma (AIS_head_ <3), coagulation abnormality and shock upon ER admission. Base excess (BE) was considered as indicator for shock [6]: BE -2 to -6 upon ER arrival was defined as moderate shock and BE < -6 as severe shock upon ER arrival. Coagulation abnormality was defined by Quick’s value ≤70%. In Germany, the prothrombin time is preferentially reported and documented as Quick’s value in percent (70-130% = normal [7]). A Quick’s value of 70% is equivalent to a INR of approximately 1.4 [8]. We have used this definition as the TR-DGU® documents global coagulation parameters (Quick’s value and PTT) only. We assumed circulatory depletion combined with coagulation abnormality in the absence of severe head injury to be most likely due to hemorrhage.

### Data analysis

Relevant clinical parameters reflecting airway management and breathing/ventilation were analyzed at scene and upon ER arrival. Support of airway (A) and breathing/ventilation (B) included intubation with mechanical ventilation and chest tube placement. Changes in oxygen-saturation (SPO_2_) were documented. The TR-DGU® does not capture ETCO2 levels into its database. Relevant laboratory parameters reflecting circulation including hemorrhage control and coagulation function were analyzed upon ER arrival and at ICU admission. The support of circulation and coagulation (C) included fluid management, cardio pulmonary resuscitation, vasopressor therapy and transfusion of blood products, e.g. packed red blood cell concentrats (pRBC), fresh frozen plasma concentrats (FFP) and other hemostatic agents. Therapeutic interventions related to the C-priority were assessed by return to reference rages of base excess and hemoglobin levels (BE > -2, Hb >7 g/dl) and restored coagulation function (Quick’s value >70% and platelets count ≥100,000/μl). All transfused FFPs were fresh frozen plasma and no thawed plasma was used. Only pRBCs and FFPs that have been transfused between ER and ICU admission were considered. Although we tried to assess patients with complete data sets only, some variables were obviously missing in some patients, which is reflected by different sample sizes in the tables shown. Incomplete documentation was observed in particular for hemostatic agents. The number of data that were available for analysis is shown for every single analysis in the corresponding tables.

### Statistical analysis

Demographic and clinical data are presented as mean and standard deviation (SD) or median with inter quartile range (IQR) for continuous variables according to the underlying distribution and as percentages for categorical variables. For continuous variables normal distribution was analysed by the Shapiro-Wilk test. To detect differences between patients groups the Student’s *t*-test or Mann–Whitney-*U*-Test was performed, depending on the underlying distribution. For categorical variables the chi-square test was used. Significance level was defined as p < 0.05. Statistics were calculated using SPSS Statistical Software Package version 18 (SPSS Inc., Chicago, USA).

## Results

A total of 517 datasets of severely injured adults derived from the TR-DGU® between 2002 and 2008 were eligible for analysis. The mean age was 42.4 years ±18.0 SD, the majority was male (72.5%, n = 374) and the mean overall injury severity as reflected by Injury Severity Score (ISS) was 35.1 points ±9.0 SD (New ISS 40.7 points ± 10.5 SD) (Table 1). The median pre-hospital time of care was 70 minutes (IQR_25-75_ 51.5-90.5). The median time period for initial diagnostic procedures and treatment in the ER was 65 minutes (IQR_25-75_ 45-98 minutes) (Table 1). The median time period from ER arrival to ICU admission including emergency operative procedures was 240 minutes (IQR_25-75_ 156-342 minutes).

**Table 1:** Basic characteristics, injury severity according to patients in the state of moderate or severe shock

	**BE -2 to -6**	**BE < -6**	**Total**
***N, (%)***	259 (50.1)	258 (49.9)	517 (100)
**Age, mean (SD)**	41.9 (18.2)	42.9 (17.7)	42.4 (18.0)
**Male, n (%)**	204 (79.1)	170 (65.9)	374 (72.5)
**ISS, mean (SD)**	33.8 (7.7)	36.3 (9.9)	35.1 (9.0)
**NISS, mean (SD)**	39.9 (10.1)	41.6 (10.9)	40.7 (10.5)
**Pre-hospital time, median min. (IQR)**	70.0 (50.0–93.0)	69.0 (52.0–90.0)	70.0 (51.5–90.5)
**Emergency room, median min. (IQR)**	66.5 (47.0–101.5)	65.0 (44.0–95.0)	65.0 (45.0–98.0)
**Time from hospital admission until ICU, median min. (IQR)**	229.5 (135.8–314.3)	250.0 (168.0–367.0)	240.0 (156.0–342.5)
**Emergency operation, n (%)**	24 (9.3)	39 (15.1)	63 (12.2)
**Operation before ICU admission, n (%)**	163 (62.9)	151 (58.5)	314 (60.7)

### Operative procedures

Diagnostic procedures and treatment in the emergency room (ER) were interrupted in 9.3% (n = 24/259) of patients with moderate shock and in 15.1% (n = 39/258) of patients with severe shock due to emergency operation e.g. laparotomy or thoracotomy. 62.9% (n = 163/259) of patients with moderate and 58.5% (n = 151/258) of patients with severe shock underwent operative procedures according to “damage control” principles after diagnostics had been completed in the ER. Patients without need for emergency or early surgical intervention were directly transferred to ICU.

### Intubation and mechanical ventilation

The vast majority of patients within both groups were intubated and mechanically ventilated at the scene prior to ER arrival (Table 2). The rate of patients on mechanical ventilation increased to 93.8% (n = 242/258) among patients with moderate shock and 96.5% (n = 246/255) in patients with severe shock during ER treatment. The frequency of cardio pulmonary resuscitation via manual chest compression was 3.5% (n = 9/254) and 9.1% (n = 23/254) according to the subgroups. Across all groups, 16.6% (n = 86/517) of the patients had received a chest tube at scene. 52.6% (n = 267/508) received a chest tube prior to ICU admission. While oxygen saturation (SpO_2_) on average was below 90% at scene, these levels had increased to 95% in the ER.

**Table 2:** Oxygen saturation, frequency of intubation of patients in the state of moderate or severe shock

	***BE -2 to -6***			***BE < -6***		
	**At scene**	**ER**	**p**	**At scene**	**ER**	**p**
**SpO**^**2**^**, mean % (SD)**	89.5 (12.1)	96.8 (5.1)	<0.001	87.9 (17.2)	94.9 (11.8)	<0.001
**Intubation, n (%)**	183/258 (71.3)	242/258 (93.8)	<0.001	200/256 (78.1)	246/255 (96.5)	<0.001
**Chest tube, n (%)**	42/258 (16.3)	133/254 (52.4)	<0.001	44/256 (17.2)	134/254 (52.8)	<0.021

Mean oxygen saturation at scene and upon ER arrival, frequency of intubation and ventilation according to the subgroups of patients in the state of moderate (BE -2 to -6) and severe shock (BE < -6) upon ER admission.

### Circulation

**Figure 1:**
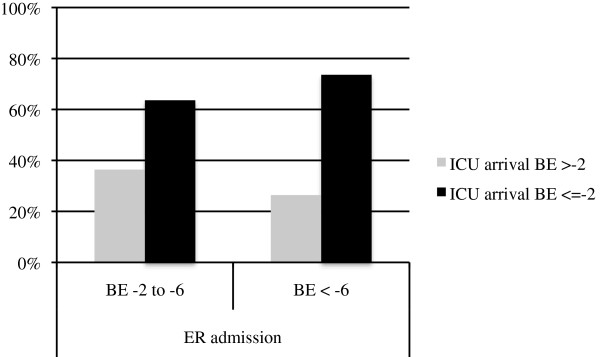
**Patients in moderate and severe shock upon hospital admission according to BE at ICU arrival.** Patients in the state of moderate (BE -2 to -6) and severe shock (BE < -6) upon ER admission and separated between those patients with physiological BE (BE ≥ -2) and decreased BE (BE < -2) upon ICU arrival.

259/517 (50.1%) patients were admitted to the ER in the state of moderate shock as reflected by BE -2 to -6 and 258 (49.9%) were in severe shock as reflected by BE < -6 (Table 1). Upon ICU admission the level of shock was reversed to BE > -2 in 36.4% of patients with initially moderate shock and in 26.4% of patients with initially severe shock (Figure 1). However, the majority of patients within both groups was still in circulatory depletion upon ICU admission. Circulatory support between ER and ICU arrival was administered via intravenous fluids, vasopressors and mechanical chest compression. While both groups had received comparable amounts of intravenous fluids the frequency of pharmacological as well as mechanical support was higher among patients who had arrived to the ER in severe shock. Table 3 provides an overview of these measures for the two groups.

**Table 3:** Fluids, vasopressors, chest decompression of patients in the state of moderate or severe shock

	**ER**	
	***BE -2 to -6***	***BE < -6***
**Crystalloids, median ml (IQR)**	2000 (1000–3500)	2000 (1000–4000)
**Colloids, median ml (IQR)**	1000 (500–1500)	1000 (500–2000)
**Catecholamine, n (%)**	123/254 (48.4)	177/254 (69.7)
**CPR, n (%)**	9/254 (3.5)	23/254 (9.1)

Fluid administration, vasopressors and cardiopulmonary resuscitation according to the subgroups of patients in the state of moderate (BE -2 to -6) and severe shock (BE < -6) upon ER admission.

### Hemorrhage and coagulation upon ER arrival

Per definition, all patients suffered from disturbed coagulation function upon ER arrival as reflected by a Quick’s value ≤ 70% (Table 4). 32.5% (n = 85/259) of patients with moderate shock and 46.5% (n = 120/258) of patients with severe shock presented to the ER with a Quick’s value <50% (Table 4). Platelets counts were depleted to <100.000/μl in approximately 10% of patients within both groups. Median hemoglobin levels were 9.7 g/dl in patients that had been moderately shocked and 8.4 g/dl in patients with severe shock. 15.5% (n = 40/258) of patients with moderate shock and 26.5% (n = 68/257) had a hemoglobin <7 g/dl upon arrival.

**Table 4:** Coagulation abnormalities upon ER admission and ICU arrival according to the subgroups

	***BE -2 to -6***			***BE < -6***		
	**ER admission**	**ICU arrival**	**P**	**ER admission**	**ICU arrival**	**p**
**Hb, median (IQR)**	9.7 (7.7–11.4)	9.1 (7.9–10.7)	0.093	8.4 (6.9–10.3)	9.6 (8.3–11.0)	<0.001
**Hb <7 g/dl, n (%)**	40/258 (15.5)	34/254 (13.4)	0.055	68/257 (26.5)	27/248 (10.9)	<0.001
**Quick’s value, median** **(IQR)**	56.1 (44.0–64.5)	60.0 (51.5–70.0)	<0.001	51.0 (41.0–61.0)	58.0 (47.8–71.3)	<0.001
**Quick’s ≤70%, n (%)**	259 (100)	188/249 (75.5)	-	258 (100)	176/240 (73.3)	-
**Quick’s ≤50%, n (%)**	85/259 (32.8)	59/249 (23.7)	0.003	120/258 (46.5)	80/240 (33.3)	0.008
**Platelets, median (IQR)**	168,000 (128,000–260,600)	98,000 (70,500–141,000)	<0.001	166,500 (120,000–214,000)	85,500 (54,000–130,500)	<0.001
**Platelets <100,000, n(%)**	29/255 (11.4)	127/244 (52.0)	<0.001	32/253 (12.6)	136/234 (58.1)	0.002

Coagulation abnormalities upon ER admission and ICU arrival according to the subgroups of patients in the state of moderate (BE -2 to -6) and severe shock (BE < -6) upon ER admission.

### Management of hemorrhage and coagulation

Attempts to correct coagulopathy upon ER arrival included the transfusion of fresh frozen plasma (FFP), platelets concentrates, fibrinogen, prothrombin complex concentrates (PCC), packed red blood cell concentrates (pRBC), recombinant factor VIIa, antifibrinolytics and other procoagulant agents (Table 5). The vast majority of patients had received at least one pRBC between ER and ICU admission. Massive transfusion (≥10 units of pRBCs) was initiated in 22.6% (n = 58/257) of patients with moderate shock and in 42.2% (n = 109/258) with severe shock. FFP concentrates were administered in 56.4% and 68.2% of the patients, respectively. The mean number of blood products as well as the distribution of factor replacement and antifibrinolytics administered to both groups are summarized in Table 5. Platelets concentrates were only transfused in 17.9% and 36.0% of the patients and if administered at all only in very low quantities (Table 5).

**Table 5:** Blood products and hemostatics according to patients in the state of moderate or severe shock

	**ER**	
	***BE -2 to -6***	***BE < -6***
**pRBC transfusion, n (%)**	178/257 (69.3)	207/258 (80.2)
**pRBC mass transfusion ≥10, n (%)**	58/257 (22.6)	109/258 (42.2)
**pRBC, median (IQR)**	4.0 (0–8.0)	7.0 (2.0–16.5)
**FFP transfusion, n (%)**	145/257 (56.4)	176/271 (68.2)
**FFP, median (IQR)**	3.0 (0–7.11)	6.0 (0–14.3)
**FFP:pRBC ratio**	0.95	0.80
**Platelet concentrates, n (%)**	46/257 (17.9)	93/258 (36.0)
**Platelet concentrates, median (IQR)**	0 (0–0)	0 (0–1.8)
**Platelet concentrates, mean (SD)**	0.4 (1.2)	1.3 (3.9)
**Fibrinogen, n (%)**	40/171 (23.4)	62/176 (35.2)
**PPSB, n (%)**	30/171 (17.5)	42/176 (23.9)
**Recombinant factor VIIa, n (%)**	9/171 (5.3)	16/176 (9.1)
**Antifibrinolytics, n (%)**	13/171 (7.6)	21/176 (11.9)
**Others, n (%)**	21/171 (12.3)	32/176 (18.2)

Transfused packed red blood cell concentrates (pRBCs), fresh frozen plasma (FFP) and hemostatics according to the subgroups of patients in the state of moderate (BE -2 to -6) and severe shock (BE < -6) upon ER admission.

### Hemorrhage and coagulation upon ICU admission

Upon ICU admission at median 240 minutes after ER arrival 3 out of 4 patients in both groups still suffered from a disturbed coagulation function as reflected by a Quick’s value ≤70%. Severe coagulation dysfunction (Quick’s value <50%) was still present in 23.7% of patients with moderate and 33.3% of patients with severe shock. Simultanously, the number of patients with platelets <100,000/nl had increased 5- to 6-fold between ER and ICU admission (Table 5). Median platelet counts had dropped from 168,000/μl to 98,000/μl in the group with initially moderate shock and from 166,500/μl to 85,500/μl with initially severe shock during the same interval. The median hemoglobin levels had increased in the group with initially severe shock only.

## Discussion

The present study evaluated in how far ABC priorities (A: airway maintenance, B: breathing/ventilation and C: circulation with hemorrhage control) with focus on the C-priority are addressed during early in-hospital care and to what extent these key issues have been controlled for prior to ICU admission in patients arriving at the ER in states of hemorrhagic shock. This analysis was based upon datasets of severely injured patients derived from the TR-DGU® database.

Patients arrived approximately 70 minutes after injury and initial pre-hospital treatment at the trauma bay. Despite improvements in the management of the severely injured at scene and a trend towards faster transportation to an appropriate medical facility, the “golden hour”, as advocated by R.A. Cowley and colleagues already in the late 1970s, was still not matched in most of the patients studied here [9]. The “golden hour” is a widely accepted term emphasizing the relevance of time in trauma care. Liberman and co-workers have conducted a meta-analysis comparing advanced versus basic life support strategies and showed that a prolongation of pre-hospital resuscitation was associated with significantly increased mortality [10]. However, “definitive care” for time critical issues such as ABC can be delivered under particular circumstances by highly trained pre-hospital teams within the golden hour. Therefore, the golden hour does not necessarily be missed despite pre-hospital rescue times exceeding one hour.

In the present study, a safe airway and controlled ventilation was established in 71- 78% of patients during the early pre-hospital phase of care according to the subgroups. Procedures like intubation and mechanical ventilation are associated with potential risks to the patient, for example, if not performed appropriately this may result in hypoxia including neurological consequences. Furthermore, the time factor needs to be considered which may delay transport to the appropriate hospital. Therefore, such interventions should be restricted if really needed and only to secure airway and assure ventilatory support [11].

The median time window in the ER for assessment and management according to ATLS and diagnostic procedures was 65 minutes. This time period included interventions such as iv-lines, chest tubes, wound management and diagnostics, for example focused assessment with sonography for trauma (FAST), x-ray, CT and cCT scaning. Huber-Wagner and colleagues emphasized whole body CT-scanning upon ER arrival as a fast and comprehensive diagnostic tool for early detection of injuries which was associated with an increased survival rate [12]. Diagnostic procedures in the ER were interrupted in 9.3% of patients with moderate and in 15.1% of patients with severe shock due to emergency operations. The majority of severely injured patients were treated by damage control principles of surgery. Surgical procedures were addressed to life-threatening injuries expediently, while definitive surgical care was followed after stabilisation and restored physiology [13]. Overall, the mean time interval from ER arrival until ICU admission including operative procedures was 240 minutes.

An interesting finding of the present study was that despite resuscitation efforts including damage control principles, fluid administration, circulatory and coagulation support during the early phase of in-hospital management, shock was reversed in only 1 out of 3 patients with initially moderate and in only 1 out of 4 patients with initially severe shock upon ER arrival. Thus, the majority of patients in the present study was still in the state of shock when admitted to the ICU. Although not strictly documented in the TR-DGU® these patients were frequently acidotic upon ICU arrival. Acidosis has frequently been discussed as an important trigger for coagulation dysfunction after trauma [14,15] representing also a major component of the so-called “lethal triad” including hypothermia, acidosis and coagulpathy [16].

In the present study a mean of 3 litres of fluids including crystalloids and colloids were administered during ER treatment and diagnostics. Hussmann and colleagues have conducted a retrospective study of matched pairs including 1896 severely injured patients (ISS ≥ 16) with fluid administration ≤1,5 L vs. >1,5 L during the pre-hospital phase of care. Patients with fluid administration >1,5 L had a significantly higher need for blood product transfusion and a reduced ability to clot [17]. Trauma patients with increased administration of fluids had also a significantly higher mortality (low-volume: 22.7%, high-volume: 27.6%; p < 0.01). Maegele and co-workers screened datasets of 8724 trauma patients from the TR-DGU® and showed an increase in the frequency of coagulopathy with increased amounts of fluids administered [18]. In this study coagulopathy was present in >40% of patients with >2 L of fluids administered, in >50% with >3 L, and in >70% with >4 L fluids. The current literature and resuscitation in trauma suggests a more restrictive use of intravenous crystalloids and colloids but advocates the early use of blood products as the appropriate means of correcting hypovolemia. Vasopressors are currently not considered as a suitable approach for addressing acute hypovolemia and have been associated with increased mortality in some studies [19].

Another important observation from the present study was that initially disturbed coagulation function present in all patients upon ER arrival per definition could not be restored in 3 out of 4 patients prior to ICU admission. Moreover, median platelet counts had dropped to values below 100.000/μl upon ICU arrival and the percentage of patients with significant thrombocytopenia had increased from 10% to >50% during the same time interval. Previous studies have shown that disturbances in coagulation function after trauma especially in combination with hypoperfusion secondary to shock may dramatically increase mortality [20]. Unfortunately, these drops in platelet counts could not be viewed in the context of platelet function as this information is not captured in the TR-DGU®.

In the present study, the majority of patients had received at least one pRBC between ER and ICU arrival and massive transfusion was initiated in 23% and 42% according to the subgroups with moderate and severe shock. In contrast, FFP concentrates were administered to a lesser extent and platelet concentrates were only transfused in 18% and 36% of patients, respectively, and if administered at all only at very low quantities. This is in contrast to recent evidence from the literature indicating a survival benefit if coagulation abnormalities after trauma are addressed aggressively from the very moment on as the bleeding trauma patient hits the ER door [3-5,21-23]. Previous work from our group together with work from others has shown that mortality from trauma hemorrhage can be reduced by more balanced transfusion strategies involving pRBC and FFP transfusion in more equal ratios. Holcomb and colleagues extended this strategy by adding platelets to this approach suggesting the balanced administration of FFP: pRBC: platelets in 1:1:1 ratios [5]. In our patient cohort presented here platelet counts dropped significantly between ER arrival and ICU admission and more than half of all patients presented with significant thrombocytopenia upon ICU admission. According to our analysis, the administration of platelet concentrates was a rarity at least in the setting assessed here. Therefore, severely injured patients even with a normal platelet count upon ER arrival seem to have a high risk to develop a thrombocytopenia and should therefore be assessed for platelet counts sequentially. Vice-versa, the use of blood products including massive transfusion has frequently been shown to be associated with risks, for example single and multi organ failure. However, the hazards of transfusion may appear somewhat trivial relative to the need of care for an exsanguinating patient.

Damage control resuscitation by using pRBC and FFP only, as advocated now by many authors, may be too time consuming to reverse global coagulation parameters into reference ranges in adequate time windows. Gonzales and colleagues reported time windows up to 14.2 hours to set back global coagulation parameters into reference ranges by using balanced ratios. Alternative approaches currently under debate suggest either the use of freezed-dried lypholized plasma or the administration of coagulation factor concentrates using an early and individualized goal directed approach to treat trauma-induced coagulopathy [24-28]. These strategies are based upon whole-blood viscoelastic testing offering a faster and more comprehensive insight into the individual coagulation in trauma including initiation, speed and quality of the clotting process [28-30]. By using this technology, it is possible to differentiate different types of coagulation dysfunction e.g. hypo-/hypercoagulable coagulopathy or hyperfibrinolysis [31]. In response to the underlying coagulation dysfunction different but targeted therapeutic strategies have been discussed to stabilize coagulation function and reduce the need of blood products [26,32]. The use of viscoelastic methods apart from global coagulation tests have recently been endorsed by the updated European guidelines for the management of the bleeding follow major trauma, in which Roissant and co-workers also recommend that monitoring and measures to support coagulation in the acute bleeding situation should be initiated as early as possible [6]. The recently published S3 guideline for the management and resuscitation of severely injured patients emphasizes the central role of hemorrhage control including aggressive management of coagulation function [33].

The present report is limited by the number of included patients and its retrospective design. Another limitation is that the focus of data collection into the TR-DGU® is targeted to the first 48 hours after admission with data quality heavily weighted towards the time window between ER and ICU admission. Therefore, we are not able to report on blood product transfusion afterwards. Furthermore, the TR-DGU® does not offer more detailed information with regard to hemodynamics, coagulation management and ventilation as the data presented here. In addition, important triggers to further aggravate the acute coagulopathy of trauma, for example hypothermia, are likewise not documented into the TR-DGU®. Further research should be conducted with larger patient numbers and by using a prospective and more detailed approach to prove these results.

## Conclusions

While airways and ventilation appear to be well managed prior to ICU admission, shock was only reversed in 1 out of 3 patients with initially moderate and in 1 out of 4 patients with initially severe shock upon ICU arrival. Coagulation function could not be restored in 3 out of 4 patients prior to ICU admission. Moreover, median platelet counts had dropped to values <100.000/μl upon ICU admission and the percentage of patients with significant thrombocytopenia had increased from 10% to >50%. These potentially life-threatening issues should be addressed more aggressively in the early care of the severely injured patient.

## Abbreviations

ICU: Intensive care unit; ER: Emergency room; TR-DGU: TraumaRegister DGU®; ISS: Injury severity score; BE: Base excess; ATLS: Advanced trauma life support; AIS: Abbreviated Injury Scale; SPO_2_: Oxygen-saturation; pRBC: Packed red blood cells; FFP: Fresh frozen plasma; Hb: Hemoglobin; SD: Standard deviation; NISS: New injury severity score; IQR: Inter quartile range; PCC: Prothrombin complex concentrates; CPR: Cardio pulmonal resuscitation; min: Minute; PPSB: Prothrombin complex; Thx: Thorax.

## Competing interests

There are no competing interests associated with this article.

## Authors’ contributions

SP and MM conceived the study, SP undertook the statistical analysis together with MM and PT, all other authors (BT, MB, PS, BB) contributed to the study design and to data sharing. SP and MM signed responsible for writing the manuscript. All authors read and approved the final manuscript.
